# *Eimeria*-Induced Gut Microbiota Dysbiosis and Probiotic-Mediated Control of Chicken Coccidiosis: Recent Advances and Future Perspectives

**DOI:** 10.3390/ani16172703

**Published:** 2026-08-31

**Authors:** Kun Xu, Huricha Bao, Jianping Tao, Nianyu Xue

**Affiliations:** 1 College of Veterinary Medicine, Yangzhou University, Yangzhou 225009, China; 19962579316@163.com (K.X.); jptao@yzu.edu.cn (J.T.); 2 College of Electrical, Energy and Power Engineering, Yangzhou University, Yangzhou 225009, China; 3 School of Journalism and Communication, Yangzhou University, Yangzhou 225009, China; bao@yzu.edu.cn; 4 Jiangsu Co-Innovation Center for Prevention and Control of Important Animal Infectious Diseases and Zoonoses, Yangzhou University, Yangzhou 225009, China

**Keywords:** chicken, coccidiosis, gut microbiota, dysbiosis, probiotic

## Abstract

Avian coccidiosis is an intestinal disease caused by several different *Eimeria* spp. that impair the host’s intestinal system, resulting in poor nutrition absorption, reduced growth, and often death. Increasing evidence from recent studies indicates that *Eimeria* infection disrupts epithelial integrity and reshapes the intestinal microbial ecosystem. Probiotics can enhance poultry productivity and gut health by modulating the microbiome, with certain probiotics demonstrating anticoccidial properties. A detailed understanding of *Eimeria*-induced dysbiosis, barrier injury, mucosal inflammation, secondary bacterial risk, and probiotic-mediated control is necessary for the effective application of probiotics in commercial practice and for developing logical strategies to control coccidiosis in the post-antibiotic era. This review differs from general coccidiosis or probiotic reviews by integrating evidence across five linked dimensions: *Eimeria* species, intestinal site, time after infection, probiotic strain or function, and production translation. It summarizes the current understanding of *Eimeria*-driven gut microbiota dysbiosis and probiotic-based interventions for chicken coccidiosis, with a focus on intestinal site specificity, temporal microbial patterns, microbial metabolism, host–microbiota interactions, and practical implications for poultry production. Future research should move from correlation-based microbiota profiling toward strain-resolved, mechanism-oriented, and production-relevant evaluation.

## Simple Summary

Avian coccidiosis is one of the most economically important diseases caused by *Eimeria* spp. Gut microbiota is an important part of organisms and is closely related to the parasite and host. Probiotics have an important effect on *Eimeria*, and also help in promoting the growth of beneficial gut microbiota. This review summarizes the current understanding of *Eimeria* infection and the chicken intestinal ecosystem, the patterns of gut microbiota dysbiosis induced by *Eimeria*, the mechanisms by which dysbiosis affects intestinal health, probiotic-mediated control of chicken coccidiosis, and the mechanisms of probiotic action. It also explores the limitations of current research, evidence gaps, and future research strategies.

## 1. Introduction

Coccidiosis is a major protozoal disease that severely affects livestock and other animals, especially poultry. It is generally used to refer to infection by *Eimeria* [1], although it can broadly describe any disease caused by coccidia [2]. Although *Eimeria* spp. infects nearly all domestic animals and wildlife that have been investigated, causing a significant economic burden on commercial cattle and sheep production, coccidiosis is commonly considered to be most significant in chicken industry [3,4]. This is due in part to the vast number of chickens produced worldwide each year, their short generation time, and the narrow profit margins associated with their production [5]. Therefore, this review focuses on coccidiosis in chickens.

Chicken coccidiosis is one of the most important enteric diseases and has been ranked as the top disease concern by members of the Association of Veterinarians in Broiler Production (AVBP) in annual surveys [6], with estimated annual losses exceeding £10.36 billion [7]. Currently, coccidiosis control has relied on anticoccidial drugs, live vaccines, and management practices that reduce oocyst accumulation in litter. These preventive measures have enabled intensive poultry production, but their limitations are becoming increasingly apparent. Although rotation and shuttle programs are employed to delay the development of resistance, continuous use of ionophores and synthetic coccidiostats may still lead to the formation of resistant strains [8,9]. Live vaccines can induce protective immunity and may help restore drug sensitivity in field populations, yet their efficacy depends on uniform administration, oocyst cycling, litter conditions, bird age, and the balance between early parasite replication and acceptable performance loss [10,11]. Management practices such as litter control, stocking density management, and hygiene reduce exposure pressure but cannot fully prevent infection in commercial systems. These limitations support integrated programs that combine drugs or vaccines with nutrition- and microbiota-targeted interventions [6,9]. Therefore, much effort has been made to develop alternative strategies, including dietary strategies using phytochemicals, probiotics, prebiotics, and others.

Traditionally, seven *Eimeria* species, including *Eimeria tenella*, *E. necatrix*, *E. acervulina*, *E. maxima*, *E. brunetti*, *E. praecox*, and *E. mitis*, are recognized to infect chickens, each with a specific intestinal site and distinct pathogenic characteristics [12]. *Eimeria* parasites multiply in the intestines of chickens and cause tissue damage, lowered feed intake, poor absorption of nutrients from the feed, dehydration, and blood loss [13]. Currently, an increasing number of studies have shown that *Eimeria* infection leads to intestinal microbiota disturbance and intestinal dysfunction. Dysbiosis may participate in the disease process by weakening colonization resistance, reducing short-chain fatty acid-producing bacteria, altering mucosal immune tone, and increasing epithelial permeability, and could promote the establishment and growth of potentially pathogenic bacteria [14,15,16,17]. Therefore, maintaining balanced gut health and improving the gut health of chickens are important strategies for controlling coccidiosis.

Probiotics, as live nonpathogenic microorganisms, have the potential to maintain a normal gut microbiota through diverse modes of action, including competitive exclusion, production of antibacterial compounds, stimulation of the immune system, enhancement of gut barrier integrity and reduction in the severity of intestinal lesions. They are widely used in the poultry industry as natural alternatives to traditional chemotherapeutic drugs [18,19,20], with some probiotics exhibiting anticoccidial properties. Studies using *Bacillus*, *Lactobacillus*, *Clostridium butyricum*, and multi-strain preparations suggest potential benefits for growth performance, lesion scores, oocyst shedding, intestinal barrier function, mucosal immunity, antioxidant status, and microbial recovery under *Eimeria* challenge [21,22,23,24]. However, a detailed understanding of *Eimeria*-induced dysbiosis, barrier injury, mucosal inflammation, secondary bacterial risk, and probiotic-mediated control is required for the effective application of probiotics in commercial practice. This paper will therefore provide an overview of current knowledge regarding *Eimeria*-driven gut microbiota dysbiosis and probiotic-based interventions for chicken coccidiosis, with a focus on intestinal site specificity, temporal microbial patterns, host–microbiota interactions, and practical implications for poultry production. Furthermore, this review differs from general coccidiosis or probiotic reviews by integrating evidence across five linked dimensions: *Eimeria* species, intestinal site, time after infection, probiotic strain or function, and production translation. This structure is intended to separate site-specific microbial associations from intervention-linked findings and to identify where causal validation and field-scale evidence are still needed.

## 2. Methods

This narrative review was conducted using a structured and reproducible literature search and synthesis strategy. Searches were performed in PubMed, Web of Science Core Collection, Scopus, ScienceDirect, and CAB Abstracts on 10 July 2026. The search covered the literature published up to 10 July 2026, with emphasis on studies published from 2020 to 2025 and inclusion of earlier foundational studies when necessary. The main search concepts were chicken coccidiosis, *Eimeria* infection, gut microbiota or microbiome, intestinal dysbiosis, intestinal barrier function, mucosal immunity, probiotic or direct-fed microbial intervention, and commercial production relevance.

The core search strings were: (“*Eimeria*” OR “coccidiosis”) AND (“chicken” OR “broiler” OR “poultry”) AND (“microbiota” OR “microbiome” OR “dysbiosis”); (“coccidiosis”) AND (“probiotic” OR “direct-fed microbial” OR “Bacillus” OR “*Lactobacillus*” OR “*Enterococcus*” OR “*Saccharomyces*”) AND (“broiler” OR “chicken”); (“*Eimeria*”) AND (“intestinal barrier” OR “tight junction” OR “mucosal immunity” OR “cytokine”) AND (“chicken”); and (“*Eimeria*” OR “coccidiosis”) AND (“metagenomics” OR “transcriptomics” OR “metabolomics” OR “multi-omics”) AND (“chicken” OR “broiler”). The number of records retrieved for each search string in each database is provided in Supplementary Table S1.

Records were screened first by title and abstract, followed by full-text assessment. Studies were prioritized when they involved chickens, experimentally or naturally relevant *Eimeria* infection, gut microbiota or intestinal health outcomes, probiotic or direct-fed microbial interventions, classical coccidiosis endpoints such as lesion score or oocyst shedding, or multi-omics analyses relevant to host–microbiota interactions. Studies were excluded when they focused on non-avian hosts, non-*Eimeria* protozoa, unrelated intestinal diseases, and non-probiotic feed additives without microbiota or intestinal-health endpoints, or lacked sufficient methodological detail. Reference lists of relevant reviews and primary studies were also checked manually to identify additional eligible articles.

Because this article is a narrative review rather than a formal systematic review or meta-analysis, evidence was synthesized descriptively rather than pooled quantitatively. Greater weight was given to chicken in vivo studies, strain- or product-defined probiotic interventions, studies reporting both classical anticoccidial endpoints and microbiota or host-response outcomes, replicated experimental designs, and field-relevant evidence. When studies produced inconsistent findings, differences in *Eimeria* species, challenge dose, bird age and genotype, diet, probiotic strain, dose, delivery route, sampling site, and sampling time were considered before assigning the relative strength of evidence.

## 3. *Eimeria* Infection and Chicken Intestinal Ecosystem

Chicken coccidiosis is biologically diverse because it is not caused by a single parasite with a uniform intestinal niche. Several *Eimeria* species infect chickens, and the major pathogenic species differ in preferred site of development, lesion type, replication dynamics, and production impact. This site specificity is central to understanding microbiota responses because the chicken gastrointestinal tract is not microbiologically uniform. The duodenum, jejunum, ileum, and ceca differ in transit time, nutrient availability, oxygen exposure, mucosal structure, immune cell composition, and microbial density. Consequently, the same parasite burden can have different ecological consequences depending on where the parasite develops.

### 3.1. Eimeria acervulina

*E. acervulina* primarily infects the duodenum and upper small intestine, where digestion and nutrient absorption are highly active. Infection in this region is usually associated with malabsorptive disease, reduced nutrient transporter function, mucus accumulation, and impaired feed conversion. Because the upper small intestine has relatively lower microbial biomass and is often dominated by *Lactobacillus*-related taxa, even moderate epithelial disruption can alter the relationship between luminal bacteria, mucosa-associated bacteria, and nutrient flow. Campos et al. (2023) [16] showed that *E. acervulina* infection affected both luminal and mucosal microbiota of the duodenum and jejunum, with mucosal communities showing site- and time-dependent changes in alpha and beta diversity. This finding is important because mucosa-associated microbiota may be more directly involved in epithelial signaling and barrier repair than luminal communities alone [25,26].

### 3.2. Eimeria maxima

*E. maxima* is commonly associated with the mid-small intestine, especially the jejunum and ileum, and is often linked to reduced body weight gain, impaired nutrient use, and marked variation in host response. Its life cycle coincides with destruction of the intestinal mucosa, lesion development, and oocyst shedding around the acute phase of infection. Recent temporal analysis showed that *E. maxima* infection induced transient but pronounced shifts in ileal microbiota around 5 to 7 days post-infection, whereas cecal changes persisted longer, even after the ileal community began to recover [17]. This indicates that a parasite developing mainly in the small intestine can still reshape the distal microbial ecosystem, likely through changes in nutrient flow, inflammation, digesta composition, and host physiology.

### 3.3. Eimeria tenella

*E. tenella* primarily develops in the ceca and is one of the best-studied species in relation to microbiota. The ceca contain the largest and most diverse bacterial communities in chickens and are major sites for fermentation and short-chain fatty acid production. Cecal infection therefore occurs within a densely colonized microbial habitat. Severe *E. tenella* infection can cause hemorrhagic lesions, epithelial disruption, inflammatory cell recruitment, and altered mucus and immune defenses. Studies have shown that *E. tenella* infection can increase Proteobacteria and Enterobacteriaceae while reducing Firmicutes or beneficial commensals such as *Lactobacillus* and *Faecalibacterium* [14,15,27]. Because the ceca are also reservoirs for opportunistic bacteria, *E. tenella*-induced dysbiosis may help explain why coccidiosis predisposes birds to secondary enteric diseases.

### 3.4. Eimeria necatrix

*E. necatrix* infects the mid-intestine and can cause severe hemorrhagic enteritis. Although less frequently studied in microbiome research than *E. tenella*, recent work indicates that *E. necatrix* infection can link histopathological damage, microbial disturbance, and metabolic disruption. Chen et al. (2025) [18] reported dose-associated villous injury, increased opportunistic taxa such as *Shigella* and *Escherichia coli*, reduced *Lactobacillus*-related bacteria, and altered energy and amino acid metabolism after *E. necatrix* infection. This supports the broader concept that intestinal pathology, microbial ecology, and host metabolism are interconnected rather than independent outcomes.

These species-specific patterns have important methodological implications. Sampling only cecal digesta may miss key events in *E. acervulina* or *E. maxima* infection, whereas sampling only the small intestine may underestimate downstream fermentation and opportunistic bacterial shifts. Luminal and mucosal samples also should not be treated as equivalent. A site-aware framework is therefore essential for interpreting *Eimeria*-microbiota studies and for designing probiotic interventions that target the relevant ecological niche. Selected pathogenic *Eimeria* species, intestinal tropism, pathology, sampling sites, and microbiota-related interpretation points are summarized in Table 1.

## 4. Patterns of *Eimeria*-Induced Gut Microbiota Dysbiosis

### 4.1. Temporal Dynamics

Evidence from recent sequencing studies indicates that *Eimeria*-induced dysbiosis is shaped by time post-infection, intestinal segment, *Eimeria* species, lesion severity, and host background. Therefore, it is more informative to discuss microbial patterns across biological dimensions than to list individual studies sequentially. The first dimension is time. During early infection, microbial changes may be modest because parasite invasion has begun, but epithelial destruction, inflammation, and oocyst shedding are not yet maximal. In the acute phase, usually around peak intracellular replication and lesion development, community shifts become more pronounced. During recovery, some communities partially return toward baseline, whereas others remain disturbed [16,17,28]. Liu et al. (2024) [17] showed this temporal structure clearly in *E. maxima* infection: ileal Shannon diversity and evenness declined at 5 to 7 days post-infection and recovered later, while cecal changes persisted to 10 and 14 days post-infection. This suggests that small intestinal communities may respond sharply but transiently, whereas distal microbial ecosystems may retain longer effects after the peak of parasite replication. Recent high-resolution omics studies further show that the microbiota response is species- and compartment-dependent, with distinct profiles during infection and recovery and between chickens showing different resistance phenotypes [28,34,35].

### 4.2. Intestinal Site Specificity

The ceca function as microbial fermentation chambers characterized by high bacterial density and diversity, whereas the duodenum and jejunum have lower biomass, faster transit, and closer links to digestion and absorption. Accordingly, cecal studies often detect broader shifts in community composition, particularly among Firmicutes, Bacteroidetes, Proteobacteria, *Lactobacillus*, *Faecalibacterium*, *Enterococcus*, *Escherichia-Shigella*, and *Clostridium*-related taxa [14,27]. In contrast, small intestinal studies may reveal more localized changes in mucosa-associated taxa and predicted metabolic functions [16,17]. Campos et al. (2023) [16] found that *E. acervulina* altered the luminal and mucosal microbiota of the duodenum and jejunum in different ways: duodenal mucosal microbiota appeared to be affected for a longer period, while jejunal mucosal microbiota showed shorter-term disruption. The study also reported lower relative abundance of bacterial taxa associated with short-chain fatty acid production, including *Lachnospiraceae*, *Subdoligranulum*, and *Peptostreptococcaceae*. These findings emphasize that sampling site and sample type can significantly influence the biological interpretation of dysbiosis.

### 4.3. Parasite Species

For *E. acervulina*, parasite infection led to changes in alpha diversity only in the mucosal microbiota, with seemingly longer-term effects in the duodenum and shorter-term effects primarily in the jejunum. Beta diversity results were consistent with this pattern. The relative abundance of potential SCFA-producing bacteria, such as Lachnospiraceae, Subdoligranulum, and Peptostreptococcaceae, tended to be lower in *E. acervulina*-infected chickens. Predicted metagenomic functions showed that pathways related to SCFA production, especially butyrate, may be affected by these changes in relative abundance during infection [16].

*E. tenella* has been strongly associated with cecal dysbiosis. Macdonald et al. (2017) [14] reported that overall alpha diversity in the cecal microbiome remained largely stable after infection, but the abundance of specific taxa changed markedly with lesion severity. Severe pathology was associated with increased Enterobacteriaceae and decreased Bacillales and Lactobacillales. Chen et al. (2020) [27] similarly found that *E. tenella* infection reduced *Lactobacillus*, *Faecalibacterium*, *Ruminococcaceae* UCG-013, *Romboutsia*, and *Shuttleworthia*, while increasing opportunistic bacteria such as *Enterococcus* and *Streptococcus*. Choi and Kim (2022) [15] further showed a dose-related increase in Proteobacteria and decrease in Firmicutes, together with reduced secretory immunoglobulin A and goblet cell density. These studies indicate that *E. tenella*-induced dysbiosis is not always reflected by a simple loss of diversity; rather, disease relevance may lie in the expansion of opportunistic taxa and the loss of beneficial functions.

For *E. maxima*, dysbiosis appears closely linked to the acute infection phase and host recovery. Jebessa et al. (2022) [36] reported distinct differences between jejunal and cecal microbiota in indigenous chickens after *E. maxima* infection, including increased *Campilobacterota* in the jejunum and Proteobacteria in the cecum, along with shifts in *Lactobacillus*, *Helicobacter*, *Alistipes*, *Barnesiella*, and *Faecalibacterium*. Liu et al. (2024) [17] further showed that the ileal microbiota underwent a transient shift, while cecal *Faecalibacterium* declined progressively and remained reduced during recovery. Several short-chain fatty acid-producing bacteria, including *Faecalibacterium*, *Mediterraneibacter*, and *Romboutsia*-related taxa, were suppressed. In contrast, *Escherichia*, *Enterococcus*, *Lactobacillus*, *Ligilactobacillus*, and *Limosilactobacillus* exhibited transient blooms with varying kinetics. This pattern suggests that inflammation may temporarily favor facultative or aerotolerant bacteria, while reducing anaerobic fermenters that support barrier and metabolic homeostasis.

For *E. necatrix*, available evidence is more limited but increasingly important. Chen et al. (2025) [18] reported that high-dose *E. necatrix* infection shifted the microbiota away from *Lactobacillus*-dominated communities toward Proteobacteria- and Enterobacteriaceae-enriched profiles. Beneficial taxa such as *Limosilactobacillus reuteri*, *Ligilactobacillus*, and *Lactobacillus crispatus* decreased, whereas *Shigella*, *Enterococcus cecorum*, and *Escherichia fergusonii* increased. Because this study also linked villous lesions with metabolic disturbances, it suggests that dysbiosis may reflect both microbial replacement and altered intestinal nutrient availability after tissue destruction.

### 4.4. Host Background

Different chicken lines may exhibit varying susceptibility to *Eimeria* and distinct microbial responses. Du et al. (2024) [30] found that yellow-feathered broilers, Arbor Acres broilers, and Lohmann pink layers differed in susceptibility to *E. tenella* and in their baseline cecal microbiota. After infection, all lines showed broadly similar directional changes, including decreased Firmicutes and Bacteroidetes and increased Proteobacteria; however, the magnitude of these changes varied among lines. *Escherichia* increased in all infected groups, while *Lactobacillus* was depleted most strongly in yellow-feathered broilers. Broadwater et al. (2025) [37] also reported breed-specific responses to *E. maxima*, with Fayoumi M5.1 chickens showing greater resistance and distinct enrichment of several commensal bacteria associated with disease resistance. These findings caution against assuming that one microbial signature applies across all genetic backgrounds.

In summary, opportunistic or inflammation-associated bacteria, especially Proteobacteria, Enterobacteriaceae, *Escherichia-Shigella*, *Enterococcus*, and *Streptococcus*, often increase during moderate to severe infection. In contrast, beneficial anaerobic fermenters and short-chain fatty acid-associated taxa, including *Faecalibacterium*, *Ruminococcaceae*, *Lachnospiraceae*, *Mediterraneibacter*, and some *Lactobacillus*-related groups, often decline, although *Lactobacillus* responses are species-, site-, and time-dependent. Alpha diversity responses are inconsistent and should not be overinterpreted alone. Beta diversity and differential abundance, especially when analyzed by lesion score, intestinal site, and time post-infection, provide more biologically meaningful information. Overall, *Eimeria*-induced dysbiosis is best understood as a dynamic disturbance of microbial function, epithelial integrity, and mucosal immunity rather than a uniform reduction in microbial diversity. Representative studies on *Eimeria*-induced gut microbiota dysbiosis are summarized in Table 2.

## 5. Mechanistic Consequences of Dysbiosis for Intestinal Health

*Eimeria*-induced dysbiosis is important because it is not merely a taxonomic signature of infection, but a functional disturbance that can reshape epithelial integrity, inflammatory tone, nutrient metabolism, and susceptibility to secondary enteric disease. Following *Eimeria* spp. infection, parasite invasion and intracellular development initiate direct mucosal damage, whereas the altered microbial community can prolong or amplify this damage after the peak of parasite replication. Studies in *E. tenella*, *E. maxima*, *E. acervulina*, and *E. necatrix* infection models consistently show that dysbiosis is influenced by parasite species, intestinal segment, sampling site, host genotype, and infection stage rather than by a uniform loss of diversity alone [14,16,17,18]. Therefore, the biological significance of dysbiosis should be interpreted through its effects on intestinal function.

### 5.1. Impairment of the Epithelial Barrier

Replication of the *Eimeria* parasite destroys enterocytes, shortens villi, disrupts crypt-villus architecture, and exposes the mucosa to luminal bacteria and antigens. Dysbiosis may aggravate this process by reducing commensal taxa associated with epithelial repair and short-chain fatty acid production, while favoring facultative anaerobes that expand under inflammatory and oxygenated conditions. In *E. tenella* infection, cecal dysbiosis has been associated with decreased secretory IgA, reduced goblet cell density, and altered antimicrobial peptide expression, indicating that mucosal defense is weakened at both physical and immune levels [15]. Severe *E. tenella* infection further demonstrates that the cecal microbiota can contribute to acute inflammation, loss of barrier integrity, and bacterial translocation, supporting the view that dysbiosis participates in pathology rather than simply reflecting it [38]. In *E. necatrix* infection, intestinal hemorrhage and villous injury coincide with a shift from *Lactobacillus*-dominated communities toward Proteobacteria and Enterobacteriaceae, suggesting that epithelial disruption creates ecological conditions favorable to opportunistic bacteria [18].

Tight junction and mucus-layer abnormalities provide a mechanistic bridge between parasite injury and microbial amplification. Tight junction proteins such as occludin, claudins, and zonula occludens proteins maintain paracellular selectivity, while goblet-cell-derived mucins restrict bacterial contact with the epithelium. When *Eimeria* damages epithelial cells, tight junction continuity and mucus organization become compromised. Changes in lamina propria thickness should also be interpreted cautiously, as an increase does not necessarily indicate enhanced nutrient absorption and may instead reflect inflammatory cell recruitment or mucosal thickening. Loss of commensal *Lactobacillus*, *Ligilactobacillus*, *Faecalibacterium*, *Romboutsia*, and *Lachnospiraceae*-related taxa may further reduce signals that normally support epithelial renewal and mucin homeostasis [16,17,18]. Conversely, blooms of *Enterococcus*, *Escherichia*, *Streptococcus*, or other facultative anaerobes may increase epithelial stimulation and inflammatory stress. This helps explain why intestinal permeability, lesion scores, and impaired feed efficiency often persist beyond the initial parasite invasion phase.

### 5.2. Activation of Inflammatory and Mucosal Immune Responses

Protective immunity against *Eimeria* requires local cellular responses, including interferon-gamma signaling, antigen presentation, macrophage activity, and T cell-mediated control. However, excessive or poorly resolved inflammation damages the mucosa and increases nutrient loss. Dysbiosis can intensify this process by enriching bacterial groups with pro-inflammatory molecular patterns and by reducing microbial signals that support immune tolerance. In coccidiosis models, infection is commonly associated with changes in cytokines such as IFN-γ, IL-1β, IL-6, IL-10, and IL-17-related responses, reflecting simultaneous parasite control, tissue inflammation, and compensatory regulation [15,24,39]. The Th17/Treg balance is particularly relevant because Th17-associated responses contribute to mucosal defense and neutrophil recruitment, whereas Treg-associated regulation is needed to prevent excessive tissue injury. Although direct mechanistic links between *Eimeria*-induced dysbiosis and Th17/Treg shifts remain incompletely defined in chickens, available evidence indicates that the intestinal microbiota can amplify inflammatory responses during Eimeria infection, while Th17/IL-17-associated responses contribute to intestinal immunopathology [38,40].

### 5.3. Metabolic Disruption

A stable intestinal microbiota contributes to fermentation, short-chain fatty acid production, bile acid transformation, amino acid metabolism, and competitive nutrient utilization. *Eimeria* infection repeatedly reduces bacterial groups linked to butyrate and other short-chain fatty acids, including *Faecalibacterium*, *Romboutsia*, *Mediterraneibacter*, *Lachnospiraceae*, and related anaerobes [16,17,37]. Butyrate supports colonocyte energy metabolism, tight junction expression, mucin production, and anti-inflammatory signaling. Its reduction may therefore connect dysbiosis with barrier leakage, villous atrophy, and inefficient nutrient absorption. In *E. acervulina* infection, altered duodenal and jejunal communities are especially important because these segments are central to digestion and nutrient uptake [16]. In *E. maxima* infection, changes in ileal and cecal microbial communities occur dynamically across acute infection and recovery, suggesting that metabolic disruption differs between small-intestinal and cecal ecosystems [17]. In *E. necatrix* infection, metabolomic disturbance involves energy and amino acid pathways, including glycolysis, tricarboxylic acid cycle-related metabolism, and pyruvate metabolism, which is consistent with severe tissue injury and impaired host nutrient use [18]. Bile acid metabolism is less directly characterized in chicken coccidiosis than short-chain fatty acid metabolism, but it is likely relevant because bile acids shape microbial composition, epithelial signaling, lipid digestion, and inflammatory tone. Integrated microbiome–transcriptome and microbiome–metabolome studies further link these compositional shifts with host gene-expression changes and altered vitamin B, amino acid, lipid, and energy metabolism, supporting a function-level interpretation of *Eimeria*-associated dysbiosis [18,41,42].

### 5.4. Increase in Risk of Secondary Intestinal Disease

**Figure 1:**
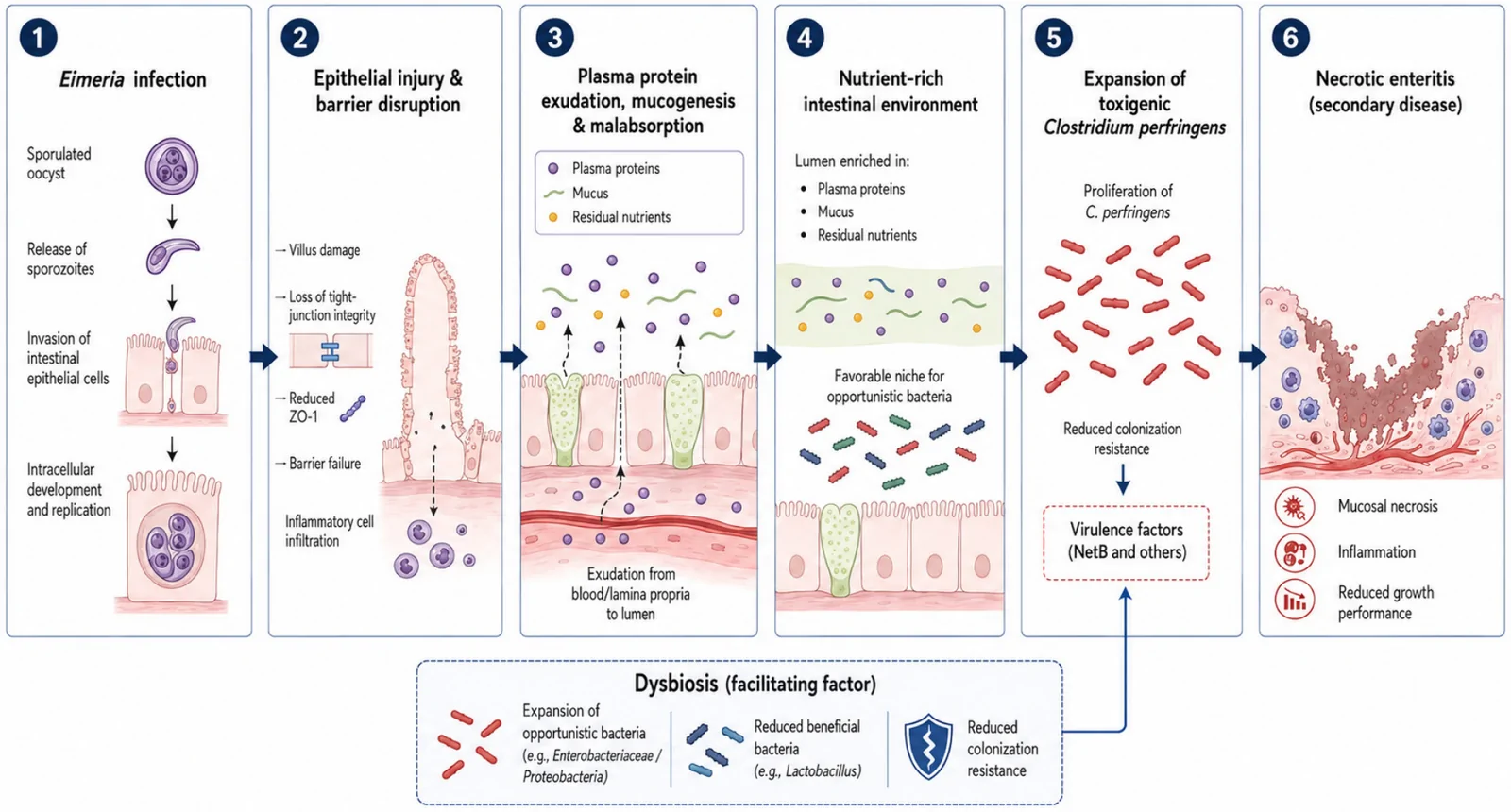
Proposed mechanism by which *Eimeria* infection predisposes chickens to secondary necrotic enteritis.

Coccidiosis is one of the most important predisposing factors for necrotic enteritis because *Eimeria*-induced epithelial injury releases plasma proteins and mucus, alters intestinal transit, increases inflammatory exudates, and creates nutrient conditions that favor *Clostridium perfringens* proliferation [43]. Dysbiosis may further facilitate the expansion of opportunistic bacteria after the parasite has damaged the mucosa [27] (Figure 1). The association between *Eimeria* challenge, Proteobacteria or Enterobacteriaceae expansion, *Lactobacillus* depletion, and barrier failure provides a plausible microbial pathway linking coccidiosis to broader enteric disease [14,27,30]. Studies using combined coccidia and *Clostridium perfringens* challenge models show that microbial interventions can improve growth and intestinal health under this dual pressure, reinforcing the concept that controlling dysbiosis is relevant not only for coccidiosis itself but also for prevention of secondary bacterial enteritis [44,45]. Thus, dysbiosis is best viewed as a mechanistic hub connecting parasite replication, epithelial damage, inflammatory amplification, metabolic inefficiency, and secondary disease susceptibility.

## 6. Probiotic-Mediated Control of Chicken Coccidiosis

Probiotic-based control of chicken coccidiosis has developed from general growth-promotion studies toward more targeted strategies that evaluate parasite outcomes, intestinal barrier integrity, mucosal immunity, and microbiota recovery. The available evidence supports probiotics as adjuncts rather than stand-alone replacements for anticoccidial drugs or vaccination. Their efficacy varies with strain identity, viability, dose, route, timing, challenge species, bird genetics, diet, and housing conditions. For this reason, probiotic studies should be interpreted at strain level whenever possible, rather than assuming that all *Bacillus*, *Lactobacillus*, *Enterococcus*, or yeast products have equivalent effects.

### 6.1. Bacillus-Based Probiotics

*Bacillus subtilis* is among the most studied probiotic species in coccidiosis-related studies. Administering *B. subtilis* has been evaluated in studies involving *E. tenella* infection, mixed *Eimeria* challenge, *E. acervulina* infection, and coccidia plus *Clostridium perfringens*. In a microbiota-focused study, administration of *B. subtilis* altered the gut microbial response to *E. tenella* infection, increasing beneficial commensal groups and partially alleviating infection-associated intestinal disruption [23]. In broilers challenged with *E. acervulina*, *B. subtilis*-cNK-2 was administered either by gavage at approximately 1 × 10^10^ CFU per bird per day or through feed at approximately 1 × 10^10^ CFU/kg, with comparison to monensin. The oral-gavage approach improved growth performance, gut integrity, mucosal immune responses, and microbiome profiles after challenge, with several outcomes approaching the anticoccidial control [24]. These results suggest that route and delivery precision matter, particularly during the narrow window around parasite invasion and mucosal injury.

*B. subtilis* has also been tested with vaccination. In Mahuang chickens, *B. subtilis* combined with a live coccidiosis vaccine improved growth indices and reduced lesion scores and oocyst output compared with infected controls, while shifting the intestinal microbiota toward genera such as *Romboutsia*, *Blautia*, and *Butyricicoccus* [46]. This is important because the formation of vaccine-induced immunity requires limited parasite replication, but excessive replication may impair growth. A probiotic that supports barrier function and microbiota stability during this window may improve the balance between immunization and performance. In yellow-feathered broilers challenged with coccidia and *Clostridium perfringens*, administration of *B. subtilis* QST713 at 100 mg/kg improved growth performance and intestinal health, showing relevance for coccidiosis-associated necrotic enteritis risk [45]. Across these studies, reported outcomes include improved body weight gain, better feed conversion ratio, reduced lesion severity or oocyst shedding, stronger barrier-related responses, modulation of cytokines, and partial microbiota restoration. However, the magnitude of benefit is not uniform across studies.

Fermented products produced by *Bacillus licheniformis*, supplied in feed at approximately 1 g/kg, improved average daily gain and increased anticoccidial index in broilers under coccidian challenge [47]. The intervention also altered cecal microbial composition, increased *Lactobacillus* abundance, and was associated with higher cecal short-chain fatty acid levels. This suggests that *B. licheniformis*-based products may act less as direct anticoccidial agents and more as modifiers of the intestinal ecosystem. Because fermented products contain bacterial cells, fermentation metabolites, enzymes, and other bioactive compounds, their effects cannot always be attributed to the organism alone. Future work should separate live-cell effects from postbiotic and fermentation-derived effects, while reporting strain identity, viable counts, diet matrix, and challenge dose.

*Bacillus amyloliquefaciens* is better supported in coccidia-associated necrotic enteritis models than in direct single-*Eimeria* coccidiosis trials. *B. amyloliquefaciens* CECT 5940 has been evaluated in broilers exposed to *Eimeria* followed by *Clostridium perfringens* challenge. Under this combined enteric stress, supplementation improved markers of gut integrity and immunity, including upregulation of tight junction-related genes such as TJP1 and OCLN, modulation of IFN-gamma, and increases in immunoglobulin-related responses [44]. These findings are relevant because field coccidiosis often interacts with bacterial enteritis, but they should not be overinterpreted as proof of direct anticoccidial efficacy. For *B. amyloliquefaciens*, stronger evidence is needed from trials that measure lesion scores, oocyst shedding, weight gain, feed conversion, barrier markers, cytokines, and microbiota after *E. acervulina*, *E. maxima*, *E. tenella*, or *E. necatrix* challenge.

### 6.2. Lactobacillus and Related Genera

*Lactobacillus* and related reclassified genera, including *Ligilactobacillus* and *Limosilactobacillus*, are central to evaluating the probiotic efficacy because *Eimeria* infection often reduces lactobacilli or changes their ecological balance. *Lactobacillus*-based interventions are usually delivered through feed or oral administration before challenge, although some studies use water application or continuous dietary inclusion [21,48]. In *E. tenella*-infected broilers, *Lactobacillus plantarum* supplementation at approximately 1 × 10^8^ CFU improved performance and intestinal health, reduced oocyst output and lesion scores, improved the feed conversion ratio, enhanced antioxidant enzyme responses, and upregulated barrier-related markers such as ZO and CLDN1 [39]. These outcomes indicate simultaneous effects on parasite-associated damage, epithelial integrity, oxidative stress, and immunity. In broader mixed-*Eimeria* models, probiotic supplementation through feed or water improved intestinal morphology and microflora, with route-dependent effects on lesion scores and oocyst shedding [21]. Natural microbiota studies also suggest that specific *Lactobacillus* or *Ligilactobacillus* taxa may be associated with resistance or recovery, although association does not prove that supplementation with the same taxon will reproduce protection [37].

### 6.3. Other Single-Taxon Candidates

*Enterococcus faecium* is widely used in poultry probiotic products, but direct mono-strain evidence for chicken coccidiosis is less extensive than for *Bacillus subtilis* or *Lactobacillus*. Its relevance in this review is therefore mainly as a component of multi-strain preparations or as a candidate organism requiring careful validation. *Enterococcus*-based products may contribute to competitive exclusion, lactic acid production, immune modulation, and early-life microbiota establishment. However, because *Enterococcus* includes opportunistic and antimicrobial-resistance-associated lineages, strain-level identification and safety assessment are essential. For coccidiosis studies, *E. faecium* products should be evaluated with clearly reported viable dose, delivery route, timing relative to vaccination or challenge, and outcomes including lesion score, oocyst shedding, growth, feed conversion, tight junction markers, cytokines, secretory IgA, and microbiota recovery. At present, claims for *E. faecium* should remain more cautious than for better-studied coccidiosis probiotics.

*Saccharomyces*-based interventions, including *Saccharomyces cerevisiae* and *Saccharomyces boulardii*, are also used in poultry gut-health programs. In coccidiosis contexts, the strongest evidence is often from yeast-containing combinations rather than yeast alone. *Pediococcus*-based and *Pediococcus* plus *Saccharomyces* products have been tested against *E. acervulina* and *E. tenella*, with variable effects on body weight gain, oocyst shedding, and humoral responses depending on dose and challenge species [9,49]. Yeast products may support gut health through mannan-oligosaccharides, beta-glucans, pathogen binding, immune modulation, and fermentation-related metabolites, but direct anticoccidial effects remain insufficiently established. Therefore, *Saccharomyces* should be framed as a supportive gut-health biological additive, especially in combined probiotic or synbiotic programs, rather than as a proven stand-alone anticoccidial.

### 6.4. Mixed Probiotic Preparations

Mixed probiotic preparations are particularly relevant for industry because commercial products often contain multiple bacterial and yeast species. Their advantage is functional complementarity: *Bacillus* spores provide processing stability, lactic acid bacteria support early colonization and acidification, *Enterococcus* or *Pediococcus* may contribute competitive exclusion, and yeast components may provide immunomodulatory cell-wall fractions. In experimental coccidiosis, probiotic delivery through feed or drinking water has produced route-specific effects. Continuous water application or intermittent higher-dose water programs have reduced lesion scores or oocyst shedding in mixed *Eimeria* challenge, while feed application has supported performance and intestinal morphology [50]. Combination with live coccidiosis vaccines also appears promising. In broilers receiving probiotic water programs around vaccination and challenge, the vaccine–probiotic combination improved weight gain and reduced lesion scores more effectively than either strategy alone in some comparisons [22]. These results support an integrated experimental case in which probiotics reduce the biological cost of vaccine and improve recovery from controlled *Eimeria* exposure.

Overall, probiotic-mediated control is strongest when studies connect classical anticoccidial endpoints with host and microbiota endpoints. Lesion scores and oocyst shedding remain essential, but they should be interpreted together with body weight gain, feed conversion ratio, villus morphology, tight junction expression, goblet cells, secretory IgA, cytokines, oxidative stress markers, and segment-specific microbiota restoration. The most convincing evidence comes from studies showing that probiotics reduce parasite-associated pathology while improving intestinal microbial balance, including the maintenance or enrichment of beneficial bacteria such as *Lactobacillus* and *Bifidobacterium* [21,51]. Still, heterogeneity in strains, doses, timing, and challenge models prevents simple ranking of products. Future studies should report strain accession, viable counts at feeding, delivery stability, anticoccidial program background, challenge species and dose, sampling segment, and statistical linkage between microbiota shifts and performance outcomes. The strain types, doses, routes, intervention timing, challenge models, outcomes, microbiota findings, and limitations of key probiotic studies are summarized in Table 3.

## 7. Mechanisms of Probiotic Action

The mechanisms of probiotic action against chicken coccidiosis should be separated into direct effects on the parasite and indirect effects on the host intestinal environment. Direct anticoccidial effects would include interference with sporozoite invasion, reduced intracellular development, or impaired oocyst shedding. These effects are biologically plausible for some microbial metabolites, but in vivo evidence remains limited and strain-specific. Most probiotic benefits in chickens are better explained as indirect effects: strengthening colonization resistance, improving epithelial repair, modulating inflammation, reducing oxidative stress, and accelerating restoration of the gut microbial ecosystem after *Eimeria*-induced disruption.

### 7.1. Competitive Exclusion and Niche Occupation

Probiotics introduced early in life or before *Eimeria* exposure may occupy adhesion sites, consume available nutrients, and reduce the ecological space available to opportunistic bacteria after epithelial damage. This is especially relevant because *Eimeria* infection often shifts the intestine toward facultative anaerobes such as *Enterococcus*, *Escherichia*, *Streptococcus*, and other Proteobacteria-associated groups, while reducing anaerobic commensals linked to fermentation and mucosal stability [14,17,27]. *Bacillus*, *Lactobacillus*, *Pediococcus*, *Enterococcus*, and yeast-containing products may each contribute to colonization resistance, but the effect depends on strain persistence, timing of administration, and the intestinal segment affected by the *Eimeria* species.

### 7.2. Metabolites Beneficial to Food Systems and Host Health

Probiotics have the potential to produce organic acids, bacteriocins, enzymes, and other metabolites. *Lactobacillus* and *Pediococcus* can produce lactic acid and antimicrobial compounds that reduce luminal pH and suppress some opportunistic bacteria. *Bacillus* species can produce enzymes, antimicrobial peptides, and bioactive metabolites, while fermented Bacillus products may deliver both viable cells and fermentation-derived compounds. These products do not need to kill *Eimeria* directly to improve disease outcome. By limiting expansion of secondary bacteria, supporting nutrient digestion, and shifting fermentation patterns, they can reduce inflammatory pressure during recovery. Increased *Lactobacillus* abundance and short-chain fatty acid-associated changes after supplementation with *B. licheniformis* fermentation products support this ecological interpretation [47].

### 7.3. Reinforcement of Epithelial and Mucus Barriers

Probiotic supplementation has been associated with improved villus morphology, reduced intestinal permeability, and increased expression of tight junction-related markers such as ZO, CLDN1, OCLN, and TJP1 in coccidiosis or coccidia-associated enteritis models [24,39,44]. This is mechanistically important because *Eimeria*-induced epithelial disruption allows bacterial products and luminal antigens to cross the mucosa, amplifying inflammation and impairing nutrient absorption. Probiotics may also support goblet cell function and mucus organization, although this area requires more direct histological and molecular evidence in *Eimeria*-challenged chickens.

### 7.4. Mucosal Immune Modulation

Effective control of *Eimeria* requires cellular immunity, but uncontrolled inflammation worsens tissue damage. Probiotics may help tune this balance by affecting secretory IgA, macrophage activity, T cell responses, and cytokine expression. Studies have reported probiotic-associated modulation of IFN-γ, IL-1β, IL-6, IL-10, and other immune markers after *Eimeria* challenge [24,39]. Increased IgA may improve immune exclusion at the mucosal surface, while balanced cytokine responses may preserve parasite control without excessive epithelial injury. The important point is not that probiotics simply “boost immunity,” but that they may improve the quality, timing, and resolution of mucosal responses.

### 7.5. Reduction in Oxidative Stress

*Eimeria* infection increases reactive oxygen species through epithelial injury, inflammatory cell recruitment, and metabolic stress. Oxidative damage can impair tight junctions, reduce villus function, and worsen feed conversion. *Lactobacillus plantarum* supplementation has been associated with improved antioxidant enzyme responses, including superoxide dismutase and catalase-related markers, in *E. tenella*-infected broilers [39]. *Bacillus*-based products may also reduce oxidative and inflammatory stress indirectly by improving barrier function and microbial balance. This mechanism is especially relevant in severe infection, where hemorrhage, tissue turnover, and inflammatory metabolism are pronounced.

### 7.6. Microbiota Recovery

Probiotics may shorten the duration of dysbiosis after *Eimeria* challenge by restoring *Lactobacillus*-associated stability, increasing short-chain fatty acid-producing taxa, and reducing prolonged expansion of opportunistic bacteria. In *B. subtilis* and vaccine-combination studies, improved outcomes have been linked with shifts toward *Romboutsia*, *Blautia*, *Butyricicoccus*, and other taxa associated with fermentation and gut health [46]. In studies of *B. subtilis*-cNK-2 and dietary *B. subtilis*, microbiome changes accompanied improved growth, intestinal integrity, and immune outcomes [23,24]. These findings suggest that microbiota restoration is not a secondary descriptive endpoint, but a central mechanism by which probiotics reduce the downstream cost of coccidiosis.

### 7.7. Direct Anticoccidial Effects on Parasite Development

Evidence for direct interference with the development of *Eimeria* parasite remains limited. The clearest evidence concerns sporozoite invasion and sporogony. *Lactobacillus* isolates from an Indigenous chicken significantly inhibited *E. tenella* invasion in vitro, and a later probiotic mixture also suppressed sporozoite entry into host cells [52,53]. In addition, *Bacillus* licheniformis fermented products and their surfactin fraction inhibited oocyst sporulation and altered sporozoite morphology in vitro, suggesting that probiotic-derived metabolites may reduce the infective potential of the exogenous stage [47].

Evidence for effects on schizogony and gametogony is weaker and is mostly inferred from in vivo reductions in lesion scores, oocyst shedding, and histopathological severity rather than from direct counts of meronts or gametocytes. Several probiotic or probiotic-prebiotic studies reported these indirect outcomes in *E. tenella*-infected broilers [54,55,56], but they did not directly resolve the intracellular developmental stages. Accordingly, the strongest evidence currently supports interference with sporozoite invasion and oocyst sporulation, whereas evidence for inhibition of schizogony and gametogony remains suggestive rather than definitive. Future studies should combine stage-specific histology, qPCR or RNA-based markers, and ex vivo invasion or replication assays to determine whether probiotic-associated protection reflects true developmental inhibition or predominantly host-mediated containment.

Taken together, probiotic action is best conceptualized as a layered process. Direct parasite effects are currently best supported for sporozoite invasion and oocyst sporulation, whereas most in vivo protection remains indirect and involves ecological stabilization, epithelial repair, immune regulation, antioxidant defense, and recovery of microbial metabolism. Future studies should test these mechanisms experimentally rather than infer them from performance data alone. The proposed mechanisms and relative strength of evidence are summarized in Table 4.

## 8. Current Limitations and Evidence Gaps

Despite rapid progress, evidence regarding *Eimeria*-induced dysbiosis and probiotic-mediated control remains fragmented at present.

### 8.1. High Heterogeneity of the Experimental Model

Studies differ in *Eimeria* species, inoculation dose, bird age, genetic background, housing system, diet composition, and sampling time. Some studies use single-species infection, such as *E. tenella*, *E. acervulina*, *E. maxima*, or *E. necatrix*, whereas others use mixed *Eimeria* challenge or coccidia combined with *Clostridium perfringens* to model necrotic enteritis risk [14,16,17,18,45]. These models answer different biological questions, but they are often discussed together as “coccidiosis” studies. This makes it difficult to compare microbiota responses, probiotic efficacy, lesion scores, oocyst shedding, and growth outcomes across experiments.

### 8.2. Lack of Standardization in Host and Dietary Backgrounds

Bird genotype clearly influences susceptibility to *Eimeria* infection and the magnitude of microbiota alteration, as shown by studies comparing broiler lines, layers, indigenous chickens, and genetically distinct populations [30,37]. However, many probiotic studies do not sufficiently account for host background when interpreting treatment effects. Diet is another major source of variation. Basal feed composition, protein level, fiber source, anticoccidial inclusion, enzyme use, and feed form can all influence microbial ecology and disease severity. Without detailed dietary reporting, it is difficult to determine whether probiotic effects are driven by the administered strain, the feed matrix, or interactions between diet and infection.

### 8.3. Limitations of 16S rRNA Gene Sequencing

Most microbiota studies still rely mainly on 16S rRNA gene sequencing. This approach is valuable for describing bacterial community shifts, but it provides limited resolution for strain-level identification, functional genes, virulence potential, antimicrobial resistance determinants, and metabolic pathways. Several studies have shown changes in *Lactobacillus*, *Enterococcus*, *Escherichia*, *Faecalibacterium*, *Romboutsia*, *Lachnospiraceae*, and other taxa after *Eimeria* infection, but 16S data alone cannot determine whether these taxa actively drive disease, respond passively to tissue damage, or mark broader environmental changes [16,17,27,35]. Although metabolomic and transcriptomic integration remains limited, recent studies have begun to combine host transcriptomics with gut microbiome profiles, integrate microbiome data with metabolomics, and use shotgun metagenomics to resolve functional potential beyond 16S-based taxonomic descriptions [41,42,59]. Metabolomic and transcriptomic integration remains limited. Studies such as the *E. necatrix* work linking microbiota shifts with metabolic disturbances provide a useful model, but similar multi-omics designs are still uncommon [18].

### 8.4. Insufficient Identification of Probiotic Strains and Characterization of Product Properties

Many studies report commercial products, broad bacterial categories, or genus-level names without fully documenting strain accession, viable count at feeding, stability during storage, resistance to pelleting, or survival in drinking-water systems. This is a major weakness because probiotic effects are strain-specific. For example, evidence supporting *B. subtilis*-cNK-2 cannot be generalized to all *B. subtilis* products, and findings from fermented *B. licheniformis* products cannot be attributed solely to live bacterial cells because fermentation metabolites may contribute to the response [24,47]. Similar caution is needed for *Lactobacillus*, *Enterococcus*, *Pediococcus*, and *Saccharomyces* products.

### 8.5. Deficiencies in Animal Experiment Design

Many studies have limited sample size, few pen-level replicates, or incomplete reporting of experimental units. In poultry nutrition and disease studies, the pen rather than the individual bird is often the relevant unit for performance outcomes. When microbiota, lesion score, immune markers, and performance data are collected from different subsets without clear linkage, the ability to infer biological relationships is weakened. A probiotic may improve body weight gain but show unclear microbiota effects, or it may shift microbial composition without improving feed conversion ratio. Current studies often report these endpoints separately rather than testing whether microbiota restoration mediates improvements in growth, barrier integrity, or oocyst shedding.

### 8.6. Shortage of Large-Scale Field Trials

Most evidence comes from controlled challenge studies, which are essential for mechanism, but commercial farms involve variable litter conditions, vaccine cycling, anticoccidial rotation, environmental stress, feed changes, and mixed pathogen exposure. Field studies frequently report less pronounced and more variable probiotic benefits than controlled experimental challenge models, reflecting the greater complexity of commercial production conditions. Conversely, some products may show their greatest value in reducing subclinical performance loss or necrotic enteritis risk rather than directly lowering oocyst output. Therefore, the field still lacks robust translational evidence connecting strain-defined probiotic programs with flock-level health, production economics, and reproducibility across production systems.

### 8.7. Implications for Commercial Production

Commercial relevance should be framed within integrated control programs rather than single-product efficacy. In broiler production, probiotic value depends on the background anticoccidial program, including ionophore rotation, shuttle schedules, live vaccination, litter quality, stocking density, and feed formulation. Probiotics are most likely to add value when they reduce subclinical performance loss, stabilize gut function during vaccine cycling, or lower necrotic enteritis risk under antibiotic-free production. Their effect may also depend on macronutrient balance, especially crude protein level, amino acid digestibility, fiber source, and enzyme use, as well as micronutrient sufficiency. Future trials should therefore report the full production context and include flock-level endpoints such as body weight gain, feed conversion ratio, mortality, lesion score, oocyst shedding, medication use, and economic return on investment. Although some studies have included anticoccidial drugs such as monensin as comparators, direct comparisons between conventional antibiotic-based approaches and probiotic alternatives for maintaining intestinal integrity remain scarce. Addressing these evidence gaps is essential for translating experimental findings into commercial practice.

## 9. Future Perspectives

### 9.1. Shifting from General Probiotic Supplementation to Precise Microbiome Management

Rather than asking whether probiotics broadly improve coccidiosis outcomes, studies should identify which strains, strain combinations, doses, and timing strategies are effective for specific *Eimeria* species, bird ages, host genotypes, and production systems. Precision probiotics should be selected according to defined functions, such as epithelial barrier support, short-chain fatty acid production, competitive exclusion of opportunistic bacteria, immune regulation, oxidative stress reduction, or compatibility with live coccidiosis vaccination.

### 9.2. Development of Microbial Consortia with Well-Defined Characteristics

*Eimeria* infection does not disrupt a single bacterium; it alters ecological networks across intestinal segments. Therefore, combinations of Bacillus, *Lactobacillus* or *Ligilactobacillus*, butyrate-associated anaerobes, yeast-derived components, and postbiotic metabolites may be more effective than single-strain approaches. However, defined consortia should be built from mechanistic evidence rather than product convenience. Candidate organisms should be tested for colonization ability, metabolic output, compatibility, safety, and reproducibility under *Eimeria* challenge.

### 9.3. Early-Life Intervention for Intestinal Health

The first week after hatch is a critical window for immune maturation, epithelial development, and microbiota assembly. Probiotics delivered in ovo, at hatch, through early drinking water, or during starter feeding may shape colonization resistance before birds encounter high oocyst pressure. This approach is especially relevant for antibiotic-free systems and live vaccine programs, where early gut resilience may reduce the performance cost of vaccine or natural exposure. Future studies should compare pre-challenge, during-challenge, and post-challenge administration rather than treating timing as a secondary variable.

### 9.4. Integrated Strategy for Multiple Components

Integrated probiotic, prebiotic, and postbiotic strategies also deserve more attention. Prebiotics can selectively support beneficial taxa, while postbiotics may provide stable metabolites, cell-wall components, enzymes, or immunomodulatory compounds without requiring live-cell survival. These approaches may overcome some limitations of vegetative probiotics, including pelleting sensitivity and variable colonization. Synbiotic and postbiotic designs should be evaluated with the same rigor as live probiotics, including dose, composition, stability, mechanism, and commercial practicality.

### 9.5. Shift the Focus from Correlation to Causality

Multi-omics studies are needed to shift the field from correlation to causality. Recent microbiome–transcriptome, microbiome–metabolome, and shotgun metagenomic studies provide a basis for moving from correlation toward function-resolved and strain-resolved microbiome management [35,41,42,59]. 16S sequencing should be complemented by shotgun metagenomics, meta-transcriptomics, metabolomics, host transcriptomics, and targeted immune and barrier assays. Longitudinal sampling across pre-infection, acute infection, peak lesion, oocyst shedding, and recovery phases would help identify whether microbial changes precede pathology, follow tissue damage, or mediate recovery. Causal validation could include gnotobiotic or microbiota-transfer models, defined bacterial consortia, metabolite supplementation, and pathway-specific host assays.

### 9.6. Integrating Host Genetics into Microbiome Research

Breed-specific differences in susceptibility and microbial responses suggest that probiotic efficacy may depend on host immune background and baseline microbial ecology [30,37]. Future trials should include genetically diverse birds or at least report strain, breed, sex, and source clearly. This would support more realistic recommendations for broilers, layers, local breeds, and slow-growing systems.

### 9.7. Establish a Unified Evaluation Framework

Probiotic research requires a unified evaluation framework. Core endpoints should include lesion score, oocyst shedding, body weight gain, feed conversion ratio, mortality, intestinal morphology, tight junction and mucus markers, secretory IgA, cytokines, oxidative stress indices, microbiota composition, microbial function, and economic performance. Studies should report *Eimeria* species, inoculation dose, bird age, diet, anticoccidial background, probiotic strain, viable count, delivery route, and timing. With standardized endpoints and mechanism-oriented designs, probiotic research can progress from descriptive association toward validated and transferable control strategies.

## 10. Conclusions

*Eimeria* infection induces intestinal microbiota dysbiosis, which represents an important component of chicken coccidiosis rather than a simple secondary observation. The altered microbiota interacts with epithelial barrier disruption, mucus and tight junction abnormalities, mucosal inflammation, metabolic disturbance, and susceptibility to secondary enteric disease. These effects help explain why coccidiosis causes not only visible lesions and oocyst shedding, but also impaired nutrient utilization, reduced growth performance, poor feed efficiency, and increased risk of necrotic enteritis. Probiotics have clear potential as feed additives for coccidiosis control, particularly when they improve barrier integrity, modulate mucosal immunity, reduce oxidative stress, and promote recovery of microbial functions. However, their efficacy is influenced by strain identity, dose, delivery route, timing, bird age, *Eimeria* species, diet, host genetics, and farm management. Future research should therefore prioritize standardized challenge models, strain-level product characterization, multi-omics mechanism testing, and large-scale field validation. The most useful next step is to move from describing *Eimeria*-associated microbial shifts toward proving which microbial functions can be manipulated safely, reproducibly, and economically in commercial poultry production.

## Figures and Tables

**Table 1:** Major *Eimeria* species in chickens and their intestinal tropism, pathology, and microbiota relevance.

*Eimeria* Species	Main Intestinal Tropism	Typical Pathology	Common Infection Model	Microbiota Sampling Sites	Microbiota-Related Interpretation Notes	References
*E. acervulina*	Duodenum and proximal jejunum	Whitish transverse plaques, villus blunting, impaired digestion and absorption, reduced growth	Oral gavage with sporulated oocysts; low to moderate challenge commonly used in broilers; examples include 5000 to 1 × 10^5^ oocysts depending on age and study purpose	Duodenal and jejunal digesta; duodenal and jejunal mucosa	The proximal small intestine has high oxygen exposure, rapid digesta flow, and *Lactobacillus*-dominated communities; mucosa-associated and luminal microbiota should be interpreted separately.	[12,16,28]
*E. maxima*	Jejunum and ileum, with downstream effects in the ceca	Mucoid enteritis, villus damage, malabsorption, weight-gain depression, increased susceptibility to enteric coinfections	Oral challenge with sporulated oocysts; dose varies markedly among strains because *E. maxima* isolates differ in pathogenicity	Jejunal/ileal digesta and mucosa; cecal contents for downstream fermentation effects	Ileal dysbiosis may be most evident during acute epithelial injury, whereas cecal changes can persist into recovery; SCFA-producing taxa should be interpreted in relation to sampling time.	[12,17,29]
*E. tenella*	Ceca	Hemorrhagic typhlitis, cecal cores, severe inflammation, bloody droppings, mortality in severe models	Oral gavage with sporulated oocysts; moderate to severe cecal challenge models are frequently used	Cecal contents, cecal mucosa, lesion-adjacent tissue	Cecal microbiota is dense and anaerobic; expansion of Proteobacteria, Enterobacteriaceae, *Enterococcus*, or *Streptococcus* often reflects inflammation and oxygen/nutrient shifts, but severity and time post-infection strongly affect results.	[12,27,30]
*E. necatrix*	Mid-small intestine, mainly jejunum and ileum; sexual stages involve ceca	Severe hemorrhagic enteritis, epithelial destruction, intestinal bleeding, marked growth suppression	Oral challenge models with variable high-pathogenicity isolates; often used to study severe small-intestinal injury	Jejunal/ileal lesion sites; cecal contents when systemic or downstream metabolic effects are assessed	Severe tissue damage can dominate diversity metrics; metabolic disturbance and opportunistic bacterial expansion should be interpreted with lesion severity and mortality.	[18,31]
*E. brunetti*	Lower small intestine, rectum, and cloacal region	Posterior enteritis, mucosal necrosis, impaired performance; less frequently studied than the major four species	Experimental oral infection, usually in mixed-species or comparative pathogenicity studies	Ileal, rectal, or cloacal-region samples; ceca if downstream effects are evaluated	Microbiota evidence is limited; mixed infections can mask species-specific posterior-intestinal effects.	[12,32]
*E. mitis*	Lower small intestine	Mild to moderate enteritis, reduced performance with subtle lesions	Oral single-species or mixed-species challenge; often included in vaccine or field-relevant mixtures	Ileal/lower small-intestinal contents; cecal contents in mixed models	Low lesion scores do not necessarily mean negligible microbiota effects; performance loss may occur without obvious gross pathology.	[33]
*E. praecox*	Duodenum and proximal small intestine	Usually mild gross lesions, impaired feed efficiency, reduced early performance in some models	Oral single-species or mixed-species challenge	Proximal small-intestinal digesta and mucosa	Microbiota data are sparse; effects may be underestimated when only cecal samples are collected.	[1]

**Table 2:** Summary of studies on *Eimeria* spp.-induced gut microbiota dysbiosis in chickens.

*Eimeria* Species	Chicken Breed/Age	Sampling Time	Intestinal Segment	Sequencing or Profiling Method	Main Microbiota Changes	Functional or Immune Outcomes	References
*E. tenella*	Chickens; age and breed as reported in original study	Post-challenge cecal sampling	Ceca	16S rRNA amplicon sequencing	Pathology severity was associated with cecal community shifts; severe lesions were linked with higher Enterobacteriaceae and altered Bacillales/Lactobacillales representation.	Demonstrated that cecal microbiome responses vary with lesion severity rather than infection status alone.	[14]
	Chickens; experimental *E. tenella* infection	Acute infection period	Ceca	16S rRNA amplicon sequencing	Decreases in *Lactobacillus*, *Faecalibacterium*, *Ruminococcaceae* UCG-013, *Romboutsia*, and *Shuttleworthia*; increases in *Enterococcus* and *Streptococcus*.	Linked dysbiosis with cecal inflammation and impaired intestinal homeostasis.	[27]
	Broilers; coccidiosis model	Post-infection acute phase	Ceca and cecal mucosa	16S rRNA amplicon sequencing plus mucosal immune assays	Increased Proteobacteria and reduced Firmicutes; microbial changes accompanied by altered cecal environment.	Increased cecal protein leakage, reduced secretory IgA, lower goblet cell density, and altered antimicrobial peptide responses.	[15]
	Chickens under severe cecal coccidiosis model	Acute severe infection	Ceca; barrier and inflammatory readouts	Microbiota profiling with barrier/inflammation assays	Cecal microbiota contributed to the inflammatory phenotype under severe infection.	Provided mechanistic evidence that cecal microbiota can promote acute inflammation, bacterial translocation, and loss of barrier integrity during severe *E. tenella* infection.	[38]
*E. maxima*	Chickens; dynamic infection model	Multiple time points across acute and recovery phases, including 5 to 14 dpi	Ileum and ceca	16S rRNA amplicon sequencing	Ileal diversity decreased during the acute phase and subsequently recovered, whereas cecal dysbiosis persisted longer, with reduced *Faecalibacterium* and transient expansion of *Escherichia*, *Enterococcus*, and lactobacilli during inflammation.	Highlighted different temporal windows for small-intestinal injury and cecal fermentation disturbance after *E. maxima* infection.	[17]
	Indigenous chickens; age as reported in original study	Post-infection sampling	Jejunum and cecum	16S rRNA amplicon sequencing	*E. maxima* altered gut microbiome diversity and composition in intestinal-site-specific patterns.	Supported host-background effects on microbiota responses to *E. maxima*.	[36]
	Genetically distinct chicken breeds	Before and after challenge	Ceca and intestinal immune sites	Microbiota profiling integrated with host response	Resistant birds maintained or enriched commensal taxa associated with fermentation, including *Blautia*, *Faecalibacterium*, and *Mediterraneibacter*; post-challenge *Lactobacillus* and *L. salivarius* patterns differed by breed.	Supported host genetics–microbiota interactions in resistance to *E. maxima* and identified taxa correlated with resilience.	[37]
*E. acervulina*	Ross 708 male broilers; challenged at 21 d	3, 5, 7, 10, and 14 dpi	Duodenal lumen, duodenal mucosa, jejunal lumen, jejunal mucosa	16S rRNA V3-V4 amplicon sequencing; Illumina MiSeq	Segment- and compartment-specific shifts; jejunal mucosa showed lower observed features at 7 and 10 dpi and lower Faith phylogenetic diversity at 7 dpi; duodenal lumen was distinct at 7 dpi and duodenal mucosa at 14 dpi.	Peak infection at 5 to 7 dpi coincided with community disruption; findings emphasized that luminal and mucosal microbiota cannot be pooled without losing biological resolution.	[16]
*E. necatrix*	Chickens infected with *E. necatrix*	Acute disease phase	Small intestine and ceca	Microbiota profiling with metabolomic/functional analysis	Community shifted from *Lactobacillus*-dominated profiles toward Proteobacteria and Enterobacteriaceae; increases in *Shigella*, *Escherichia coli*, and *Enterococcus* cecorum and decreases in *Limosilactobacillus* reuteri, *Ligilactobacillus*, and *Lactobacillus* crispatus were reported.	Severe lesions were associated with energy and amino acid metabolic disturbance, including glycolysis, TCA-cycle, and pyruvate metabolism pathways.	[18]
Mixed or experimental *Eimeria* infection	Broilers receiving dietary *B. subtilis* with or without coccidial infection	Post-infection sampling	Intestinal and/or cecal samples as reported	16S rRNA amplicon sequencing	Coccidial infection reduced microbial diversity, whereas *B. subtilis* supplementation partly restored commensal bacterial profiles.	Connected microbiota recovery with alleviation of intestinal disruption under coccidial stress.	[23]
*Eimeria* spp. challenge	Yellow-feathered broilers, Arbor Acres broilers, and Lohmann pink layers	Post-infection sampling	Ceca	16S rRNA amplicon sequencing	Infection decreased Firmicutes/Bacteroidetes-associated communities and increased Proteobacteria and *Escherichia*; *Lactobacillus* decreased markedly in susceptible yellow-feathered broilers.	Demonstrated that breed or line background shapes susceptibility, lesion responses, and microbiota disruption.	[30]

**Table 3:** Probiotic interventions evaluated against chicken coccidiosis.

Probiotic Strain or Product	Dose	Route	Timing	Challenge Species/Model	Main Outcomes	Microbiota Improved	Key Limitations	Reference
Fermented products produced by *Bacillus licheniformis*	1 g/kg feed	Feed	Dietary supplementation before and during coccidial challenge	Coccidial challenge model	Improved average daily gain from 21 to 35 d and increased anticoccidial index; increased cecal *Lactobacillus* and SCFA-associated responses.	Yes, cecal microbiota and SCFA profiles shifted favorably.	Fermented product contains metabolites as well as microbial components; effects cannot be assigned solely to live *B. licheniformis*.	[47]
Dietary *Bacillus subtilis*	Dose as reported in the original study	Feed	Supplementation before and during infection	Experimental *Eimeria* infection	Reduced infection-associated intestinal disruption and partly restored microbial community structure.	Yes, commensal genera increased under infection.	Controlled challenge model; mechanistic causality from microbiota to protection remains indirect.	[23]
*Bacillus subtilis* plus live coccidiosis vaccine	Dose and vaccine schedule as reported in the original study	Feed plus vaccination	Early-life vaccine and dietary supplementation	Live vaccine/challenge with *Eimeria* spp.	Reduced lesion scores and oocysts per gram; associated with *Romboutsia*, *Blautia*, *Butyricicoccus*, and quorum-sensing pathway changes.	Yes, cecal microbiota was linked with protection.	Probiotic, vaccine, and infection effects overlap; strain-specific contribution requires separation.	[46]
*Bacillus subtilis*-cNK-2	Oral gavage: 1 × 10^10^ CFU/d from d 14 to 18; feed: 1 × 10^10^ CFU/kg feed	Oral gavage or feed	Around infection; challenge at d 15	*E. acervulina*, 5000 sporulated oocysts by oral gavage	Oral treatment improved growth performance; oral and feed programs reduced oocyst shedding compared with infected controls; immune, gut-health, microbiota, and predicted functional changes were reported.	Yes, 16S and PICRUSt-based microbiome changes were analyzed.	Short experimental window; gavage delivery may overestimate practical farm effects relative to routine feed inclusion.	[24]
Probiotic products administered by feed or water	Product-specific dose as reported in the primary article or product label	Feed or drinking water	Preventive supplementation around challenge	Mixed *Eimeria* challenge including *E. acervulina*, *E. maxima*, and *E. tenella*	Improved growth performance, intestinal morphology, and selected microflora-related indices, with administration route influencing outcomes.	Partly, based on culture or targeted microbial measures.	Commercial product composition and route effects make strain-specific conclusions difficult.	[21]
*Bacillus subtilis* QST713	100 mg/kg feed	Feed	Dietary supplementation	Coccidia plus *Clostridium perfringens* or NE-associated challenge in yellow-feathered broilers	Improved growth performance and intestinal health under enteric challenge.	Microbiota-related effects reported as part of gut-health outcomes.	Mixed coccidia-NE model; conclusions apply mainly to enteric-disease mitigation rather than pure coccidiosis control.	[45]
*Pediococcus*-based commercial probiotic, MitoGrow	0.1% or 0.2% in feed	Feed	Dietary supplementation before and/or during challenge	*E. acervulina* or *E. tenella*	Improved body-weight gain and reduced oocyst output in *E. acervulina* infection; immune responses were modulated in *E. tenella* infection, with variable performance benefits.	Not comprehensively assessed by modern sequencing.	Early study; product mixture and limited microbiome resolution restrict mechanistic interpretation.	[49]
Commercial probiotic program	Product-specific dose as reported in the primary article or product label	Feed and/or drinking water	Continuous or intermittent administration	Mixed *Eimeria* challenge	Continuous water delivery reduced duodenal and jejunal lesion scores; intermittent high-dose water administration reduced oocyst shedding in some comparisons.	Not fully characterized by 16S sequencing.	Mixed probiotic and mixed challenge; direct mechanisms were not resolved.	[50]
Probiotic combined with coccidiosis vaccination	Product-specific dose as reported in the primary article or product label	Drinking water and/or feed	Administered during early life and around vaccine/challenge windows	Vaccine-associated mild challenge and subsequent *Eimeria* exposure	Combination improved weight gain and reduced duodenal lesion scores compared with vaccine alone in selected outcomes.	Not comprehensively profiled.	Vaccine interaction confounds direct probiotic effect; field validation needed.	[22]
*Bacillus amyloliquefaciens* CECT 5940	1.0 × 10^6^ CFU/g feed	Feed	Preventive supplementation	Necrotic enteritis model using *Eimeria* predisposition plus *Clostridium perfringens*	Upregulated TJP1 and OCLN, reduced IFN-gamma, increased IgA, IgG, and IgM, and improved gut integrity/immunity indicators.	Not the primary endpoint in all analyses.	NE coinfection model; does not isolate anticoccidial activity against *Eimeria*.	[44]
*Lactobacillus plantarum*	Approximately 1 × 10^8^ CFU; route-specific dose as reported in the original study	Oral or dietary administration as reported	Preventive/therapeutic supplementation around challenge	*E. tenella* infection	Reduced oocyst output, fecal score, and feed conversion ratio; improved cell-mediated and humoral immunity, antioxidant markers, PepT1, ZO, and CLDN1 expression.	Microbiota restoration was not always the central endpoint.	Broader community-level microbiota effects and strain deposition details require confirmation.	[39]

**Table 4:** Proposed mechanisms and evidence strength for probiotic-mediated control of coccidiosis.

Mechanism Category	Supporting Indicators	Representative Strains or Products	Evidence Strength ^Δ^	Main Limitations	Reference
Competitive exclusion and ecological niche occupation	Reduced expansion of opportunistic bacteria; restoration of *Lactobacillus*- or SCFA-associated taxa; altered alpha/beta diversity after infection	*B. subtilis*, *B. licheniformis* fermented products	Moderate	Most evidence is based on 16S correlations; direct colonization and exclusion of defined competitors are rarely tested.	[23,47]
Production of organic acids, bacteriocins, enzymes, and other microbial metabolites	Increased SCFA concentrations or SCFA-associated taxa; antimicrobial metabolite production; improved fermentation profiles	*B. licheniformis* fermented products and derived antimicrobial lipopeptides.	Moderate	Metabolites are often inferred rather than directly quantified; fermented products confound live-cell and metabolite effects.	[47,57]
Barrier repair and tight-junction preservation	Improved villus morphology; higher ZO, CLDN1, OCLN, or TJP1 expression; reduced permeability or bacterial translocation	*L. plantarum*, *L. plantarum* BS22	Moderate to strong	Gene-expression endpoints do not always prove functional barrier restoration; models differ in parasite species, dose, and sampling time.	[39,58]
Mucosal immune modulation	Changes in IgA, IgG, IgM, secretory IgA, IFN-gamma, IL-1beta, IL-6, IL-10, Th17/Treg-related markers, macrophage or T-cell-associated responses	*B. subtilis*, *B. amyloliquefaciens*, *L. plantarum*, probiotic–vaccine combinations	Moderate to strong	Cytokine direction depends on infection stage; immune stimulation and inflammation control are sometimes difficult to distinguish.	[24,39,48,49]
Antioxidant and epithelial stress reduction	Increased SOD1, CAT, or other antioxidant markers; reduced oxidative stress and improved epithelial repair markers	*L. plantarum*, *B. subtilis*-cNK-2	Moderate	Fewer studies include oxidative-stress readouts; antioxidant changes are usually secondary endpoints.	[24,39]
Microbiota recovery after *Eimeria*-induced dysbiosis	Partial restoration of diversity, reduced Proteobacteria/Enterobacteriaceae blooms, increased commensal or butyrate-associated taxa	*B. subtilis*, *B. licheniformis* fermented products, mixed probiotics	Strong descriptive; moderate causal	Community recovery is well described, but causality requires shotgun metagenomics, metabolomics, transplantation, or defined-consortium validation.	[23,46,47]
Anticoccidial activity and potential direct effects on parasite development	Reduced lesion scores and oocyst shedding; improved anticoccidial efficacy; occasional in vitro inhibition of parasite stages	*Lactobacillus* spp., *Bacillus* spp., *Pediococcus*-based products, *B. licheniformis* fermented products and derived antimicrobial lipopeptides	Weak to moderate	Most in vivo effects may be indirect, while direct effects on parasite development remain poorly defined.	[49,51,53,56]
Integration with vaccination, anticoccidial drugs, and management	Improved vaccine-associated performance, lower lesions under mild challenge, comparator responses with monensin or vaccine programs	*B. subtilis* plus live coccidiosis vaccine; commercial mixed probiotics; *B. subtilis*-cNK-2 with monensin comparator	Moderate	Few large-scale field trials; cost-effectiveness, product stability, and standardized dosing remain underreported.	[22,24,46]

^Δ^: Evidence strength was interpreted descriptively from chicken in vivo relevance, strain or product definition, endpoint breadth, replication, field validation, and causal testing. The grading was conservative: strong required consistent direction across multiple independent chicken challenge studies with clear strain/product identity and at least partial mechanistic or field validation; moderate indicates reproducible but incomplete or model-specific evidence; weak indicates isolated, comparator-dependent, or non-replicated effects. When studies conflicted, the lower defensible category was retained rather than averaging results.

## Data Availability

No new data were created or analyzed in this study. Data sharing is not applicable to this article. The literature sources supporting the conclusions of this review are available in the published articles cited in the reference list, and the search strategy and eligibility criteria are summarized in Supplementary Table S1.

## Supplementary Materials

The following supporting information can be downloaded at: https://www.mdpi.com/article/10.3390/ani16172703/s1, Table S1: Search strategy and eligibility criteria.
