# Supplementary material for: Eimeria-Induced Gut Microbiota Dysbiosis and Probiotic-Mediated Control of Chicken Coccidiosis: Recent Advances and Future Perspectives
Source: Animals (Basel). 2026 Aug 31;16(17):2703. doi: 10.3390/ani16172703 (PMC13565650; doi:10.3390/ani16172703)
Supplement: Supplementary file 1 [file animals-16-02703-s001.zip › animals-4499844-supplementary.pdf]

Supplementary Table S1. Search strategy and eligibility criteria

**Database**	**Search string**	**Date searched**	**Records retrieved**	**Records retained after title/abstract screening**	**Main use in review**
PubMed	(“*Eimeria*” OR “coccidiosis”) AND (“chicken” OR “broiler” OR “poultry”) AND (“microbiota” OR “microbiome” OR “dysbiosis”)	2026-08-15	187	42	*Eimeria*-induced dysbiosis
PubMed	“coccidiosis” AND (“probiotic” OR “direct-fed microbial” OR “*Bacillus*” OR “*Lactobacillus*”) AND (“chicken” OR “broiler”)	2026-08-15	134	29	Probiotic intervention
Web of Science	Same search strings as above	2026-08-16	203	38	Cross-database verification
Scopus	Same search strings as above	2026-08-16	156	31	Cross-database verification
ScienceDirect	Same search strings as above	2026-08-17	98	22	Full-text screening
CAB Abstracts	Same search strings as above	2026-08-17	71	15	Veterinary and poultry literature
